# PaFlexPepDock: Parallel Ab-Initio Docking of Peptides onto Their Receptors with Full Flexibility Based on Rosetta

**DOI:** 10.1371/journal.pone.0094769

**Published:** 2014-05-06

**Authors:** Haiou Li, Liyao Lu, Rong Chen, Lijun Quan, Xiaoyan Xia, Qiang Lü

**Affiliations:** 1 School of Computer Science and Technology, Soochow University, Suzhou, Jiangsu, China; 2 Department of Computer Sciences, University of Wisconsin-Madison, Madison, Wisconsin, United States of America; 3 Jiangsu Provincial Key Lab for Information Processing Technologies, Suzhou, Jiangsu, China; Shanghai Jiaotong University, China

## Abstract

Structural information related to protein–peptide complexes can be very useful for novel drug discovery and design. The computational docking of protein and peptide can supplement the structural information available on protein–peptide interactions explored by experimental ways. Protein–peptide docking of this paper can be described as three processes that occur in parallel: *ab-initio* peptide folding, peptide docking with its receptor, and refinement of some flexible areas of the receptor as the peptide is approaching. Several existing methods have been used to sample the degrees of freedom in the three processes, which are usually triggered in an organized sequential scheme. In this paper, we proposed a parallel approach that combines all the three processes during the docking of a folding peptide with a flexible receptor. This approach mimics the actual protein–peptide docking process in parallel way, and is expected to deliver better performance than sequential approaches. We used 22 unbound protein–peptide docking examples to evaluate our method. Our analysis of the results showed that the explicit refinement of the flexible areas of the receptor facilitated more accurate modeling of the interfaces of the complexes, while combining all of the moves in parallel helped the constructing of energy funnels for predictions.

## Introduction

Peptide-mediated interactions with proteins are important to the physiological functions of living cells [1]. Thus, structural information related to protein-peptide complexes is a rich resource for drug discovery and design [2]. There is an increasing capacity for obtaining experimental-determined structural information about protein-peptide complexes, but there is still a large gap between the requirements of pharmaceutical applications and the solved experimental structures.

Recently many papers based on physical or physical-chemical computational protein-peptide docking methods have been published. Moreover, the scoring problems and search problems are two basic and important considerations for understanding protein-peptide docking [3]. From the modeling perspective, the problem of flexibility is an un-solved problem for conventional protein docking algorithms [4].

Many studies have been conducted on computational protein docking, but most docking studies are classified into protein-protein docking and protein-ligand docking. The direct application of these methods to protein-peptide docking is not expected to provide good prediction accuracy due to the following two reasons. First, the peptide is smaller and more flexible than the docking protein. Second, the peptide is more like protein compared with a regular small molecule (ligand). Therefore, computational approach to docking proteins is an appealing alternative solution for meeting the needs. Given that about 40% of protein-protein interactions involve peptides [1], protein-peptide docking merits more specific research.

The FlexPepDock protocol was developed for refinement of coarse models of peptide-protein complex structures [5] based on the Rosetta platform [6]. This protocol only works on cases where the peptide backbone conformation within the receptor-binding site is already known. The same authors recently developed an enhanced protocol, FlexPepDock *ab-initio* (abFlexPepDock for short), to support *ab-initio* peptide folding [7]. HADDOCK was originally developed for protein-protein docking [8], [9] and was recently modified to flexible protein-peptide docking [10]. HADDOCK only treats the interface residues as possible flexible areas when docking the peptide with the receptor. Dealing with backbone flexibility in protein docking and the prediction of binding site are still an open challenge. There are many useful way to predict the binding site, like Lo et al. presented a new approach named PLB-SAVE that uses only geometrical features of proteins to predict protein-ligand binding regions [11]. Receptor flexibility and binding site prediction are also different problem. An MC-based flexible approach was reported that explicitly samples protein side chain and ligand backbone and side chain rotations was very important during protein peptide docking [12]. A molecular dynamics simulation approach, Dynadock [13], was developed for the refinement of protein-peptide complexes. However, it lacks the ability to model peptides from scratch. The PDZ-DocScheme [14] only used the peptide and protein side chains within 6 Å of the bound complex as flexible areas, whereas the rest of the protein was treated as a rigid body. A rapid sampling method based on mutually orthogonal Latin squares (MOLS) was developed to sample docking poses simultaneously during protein-peptide docking [15]. This method was also focused on the flexible peptide and ignored the flexibility of the receptor. Also there are many other methods restricted to support docking very short peptides [16], [17].

In this study, we propose a novel parallel protein-peptide docking approach that considers both *ab-initio* peptide folding and modeling of the flexible areas of the receptor. A parallel computing technique is a natural choice because of the increasing popularity of parallel computing facilities. More importantly, the parallel design proposed in this study supports our understanding of the micro behaviors when a protein docks to a peptide. During the actual docking process, there are three major behaviors: peptide folding, the docking of the receptor and the peptide, and fluctuations in the flexible areas of the receptor caused by the introduction of the folding peptide. These three movements are assumed to occur in parallel. However, existing docking approaches simulate the docking process in a serial manner. Given the simultaneous occurrence of folding and docking [7], we developed a docking method which is running in a real parallel manner.

The new method is based mainly on abFlexPepDock, but we enhanced it by using parallel computing and with flexible docking, so we refer to our method as PaFlexPepDock. We consider that PaFlexPepDock contributes significantly to the modeling of protein docking in two aspects. First, we use parallel movements to mimic the natural docking process, which suggests that the dynamical adjustments between the protein and peptide are occurring concurrently. Second, we explicitly model the flexible areas of the receptor when the protein is docking to the peptide.

## Results and Discussion

### Dataset and evaluation criteria

In this study, we developed a parallel peptide docking method based on abFlexPepDock [7] for *ab-initio* docking with a receptor that contains flexible areas. The four main procedures used for low-resolution docking (peptide folding, peptide refinement, perturbation of flexible regions in the receptor, and receptor docking with the peptide) were combined in parallel (see section Methods for details). We chose 22 unbound docking cases for our evaluation in this study.

**Figure 1:**
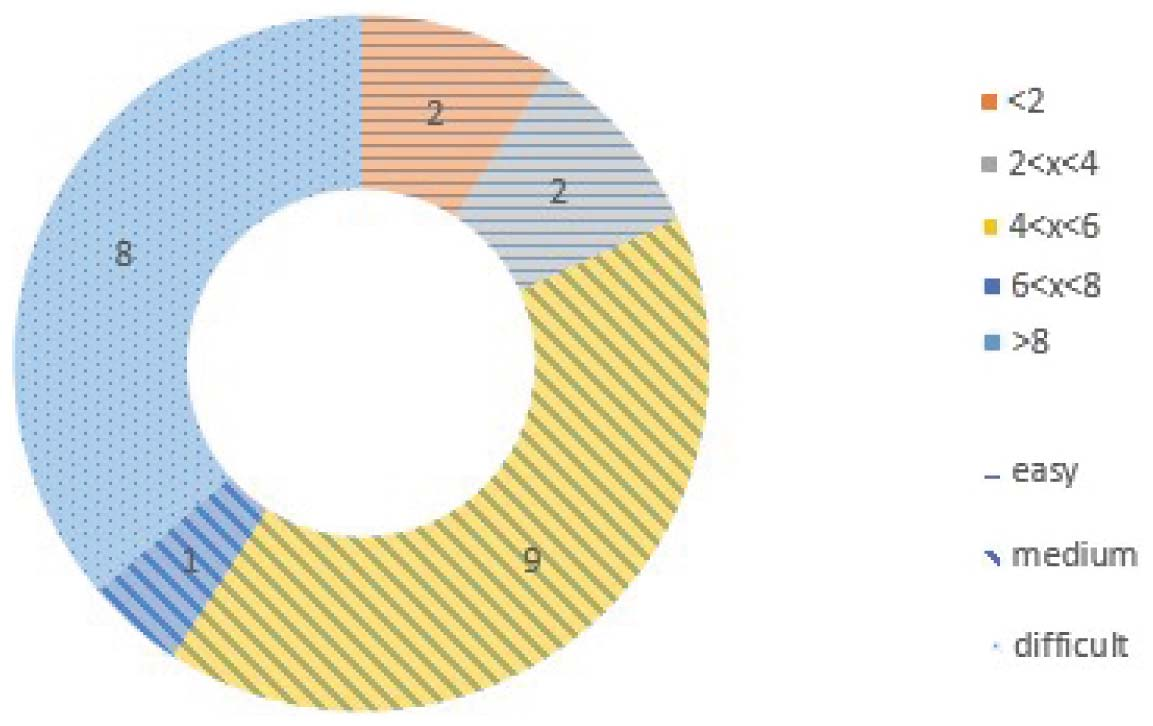
Protein-peptide test instances summary. Distribution of positional backbone RMSD between the bound form of the peptide and the ideal extended conformation.The 22 cases were divided into five levels, and classified into three categories(Easy,Medium,and Difficult).

All of the cases used in this study were chosen from the peptiDB dataset [18]. To illustrate the major differences between the unbound and bound receptor, the interface residues of unbound receptors were superimposed onto their bound counterparts using the method described in ref. [18]. These differences were measured as the C*α* RMSD (root mean square deviation) and pair (*φ*,*ψ*) deviation, respectively. As the classifying method described in ref. [10], we divided our test instances into three classes (Easy/Medium/Difficult) (see **Figure 1**).

It was based on the backbone RMSD between the conformation of the peptide in the crystal structure and its ideal extended conformation. The first eight cases (1B9K, 1JBE, 1OOT, 1R6J, 1RWZ, 2AA2, 2AM9, and 2J2I) were also considered in ref. [7] where the results were not satisfied. The two cases (1BFE and 1GFD) were taken from ref. [13] where the flexible areas of receptors were not treated explicitly. More details about these complexes can be found (see Table S1 in File S1) to assess our method.

To evaluate the accuracy of our method, we used four main general criteria: pp_bb for the peptide backbone RMSD, pep_if for the peptide backbone interface RMSD, com_if for the complex backbone interface RMSD, and com_bb for the complex backbone RMSD. All of these RMSDs were calculated after their counterparts were superimposed using the method described in ref. [18]. Like previous studies, we refered to the predictions with pep_if (≤2 Å) as near-native predictions [7] and those with pep_if (≤1 Å) as sub-angstrom predictions [5]. The prediction is said to have a successful sampling when a near-native model was generated in the final decoys.

We conducted the evaluation experiments to compare the performance of PaFlexPepDock with that of abFlexPepDock. The compared results of the first eight cases were directly adopted from ref. [7]. abFlexPepDock [7] suggested that more than 50000 decoys should be generated as the primary prediction output, and then using clustering method to identify the final predictions with good quality from the decoys. In this study, we performed PaFlexPepDock to obtain 10000 decoys as the first primary prediction results, and then followed the same clustering strategy as abFlexPepDock did to identify the final predictions. Since PaFlexPepDock used four parallel threads to explore the degrees of freedom of four various movements, these 10000 decoys could be roughly thought of as the filtered results of 40000 decoys. We think that this size of decoys is enough to get the safe conclusion not biased towards our method. In fact, we tested several cases to generate 50000 decoys (see Table S2 in File S1). The final results were not much better than those coming from 10000 decoys, but with CPU cost of almost 5 times. Thus, we decide to generate 10000 decoys for PaFlexPepDock to do the evaluation.

### Parallel performance evaluation

**Figure 2:**
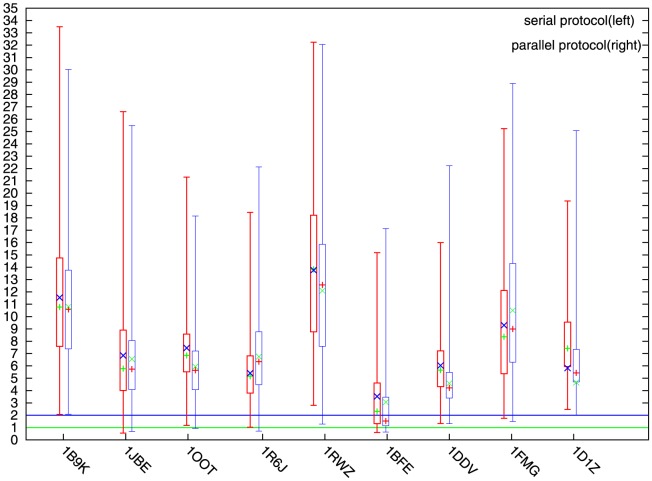
Comparison of serial and parallel running with PaFlexPepDock. Comparison of the protocol in serial and parallel mode which running with measurement pp_if. ‘+’ stands for mean value, ‘x’ stands for median value. The minimum, lower quartile, upper quartile and maximum are obviously shown without ambiguity. The green horizonal line indicates successful sampling threshold, the blue horizonal line indicates sub-angstrom sampling threshold.

First, we want to confirm that the performance of our parallel method was not worse than its own serial counterparts. We serialized the four major procedures of our parallel version of PaFlexPepDock. In order to compare with abFlexPepDock, we put the procedure of the receptor flexibility refine at the end of the main framework. So the order of the four procedures were docking, peptide abinitio, peptide refine and receptor flexibility refine. **Figure 2** shows the typical results of the comparison between the parallel and serial running of PaFlexPepDock.

The box-and-whisker plot shows clearly that there is a positive improvement for the parallel processing compared with the serial approach. There were eight successful samplings (the only exception, 1B9K, was very close to the successful sampling, i.e. 2.086 Å vs 2.000 Å) using our parallel protocol and six using the serial protocol. A comprehensive comparison of the decoys showed that in six (1B9K, 1OOT, 1RWZ, 1BFE, 1DDV, and 1D1Z) of nine cases the parallel approach was better than the serial method, particularly on 1OOT, 1DDV, and 1BFE. Therefore, it was safe to conclude that the parallel protocol improved the predictive accuracy over its serial counterpart.

Next in this study, we used the results of the parallel running of PaFlexPepDock as the representative results to evaluate its performance compared with results from the control experiments.

### Comparative analysis of results

The performance on modeling interface, *ab-initio* folding peptide backbone, modeling flexible areas and energetic ranking ability is our main concerns.

The clustering results of the docking benchmark in terms of modeling accuracy of peptide interface with PaFlexPepDock and abFlexPepDock were summarized in **Table 1**.

**Table 1:** The discriminative ability comparison between PaFlexPepDock and abFlexPepDock.

pdb_id	best pep_if†		top-10 pep_if§		first near native cluster¶	
1B9K	2.086	1.2	5.642	1.4	>500	8
1JBE	0.598	0.4	0.96	5	2	29
1OOT	0.933	1.1	1.258	3.2	3	22
1R6J	0.71	0.7	1.9	1.9	1	1
1RWZ	0.847	1.9	4.281	4.3	136	>500
2AA2	0.646	0.7	1.6	1.5	4	10
2AM9	0.362	0.7	0.794	2.6	1	28
2J2I	1.454	1.8	2.759	3.7	4	299
1I2H	1.179	1.363	2.393	1.98	1	1
1FMG	1.495	1.299	5.903	2.025	18	8
1SPR	1.732	2.79	3.887	3.054	205	>500
1Y0M	0.67	1.178	0.869	1.226	1	19
2G6F	0.724	1.459	1.372	3.774	1	25
2DS8	1.375	1.533	1.892	2.471	8	8
1BFE	0.588	0.621	1.314	1.353	1	1
1GFD	1.476	1.944	2.036	2.513	2	150
1EG3	1.789	2.219	3.922	3.603	42	>500
1GO5	1.109	1.496	2.314	3.977	1	28
1Z9L	1.096	2.735	2.984	3.331	12	>500
2YQL	0.914	0.964	1.403	1.46	1	1
1V49	1.247	1.674	3.79	4.917	191	146
1D1Z	1.658	3.18	4.086	4.208	155	>500

Cluster performance of peptide modeling onto unbound protein receptor structures. For each pair, left and right column are generated by PaFlexPepDock and abFlexPepDock respectively.

† The best pep_if among all sampled decoys.

§ The best pep_if of the representing prediction among top-10 clusters.

¶ The rank of the first cluster with near native structure.

For protocol PaFlexPepDock, we can sample near native conformation in almost all cases, where half of the cases the near native model was ranked within the top-10 ranking clusters. **Table 1** shows that our PaFlexPepDock got an obviously better result than abFlexPepDock when selecting the best prediction in terms of modeling peptide interface.

**Table 1** ensured us that our protocol PaFlexPepDock was able to identify the best models from decoys. We now moved to evaluate modeling interface combined with the consideration of the complex interface (com_if). **Table 2** shows the results in all the four criteria, including com_if.

**Table 2:** The overall comparison between PaFlexPepDock and abFlexPepDock.

	PaFlexPepDock(Å)				abFlexPepDock(Å)			
pdb_id	com_if†	pp_bb§	pep_if‡	com_bb¶	com_if†	pp_bb§	pep_if‡	com_bb¶
1B9K	1.237	2.07	2.086	1.306	0.972	1.599	1.581	1.278
1JBE	0.643	0.618	0.598	0.547	0.656	0.741	0.634	0.783
1OOT	0.716	1.737	0.933	1.128	1.03	2.8	1.467	1.478
1R6J	0.591	0.71	0.71	0.535	0.703	1.015	1.015	0.542
1RWZ	1.666	0.847	0.847	0.748	2.113	2.339	2.339	0.89
2AA2	0.449	1.088	0.646	1.387	0.396	1.644	0.532	1.414
2AM9	2.615	0.448	0.362	3.689	2.807	0.489	0.439	3.701
2J2I	0.836	1.494	1.454	0.495	0.952	1.576	1.672	0.505
1I2H	1.102	1.289	1.179	1.341	1.161	1.371	1.363	1.341
1FMG	0.994	1.495	1.495	0.71	0.898	1.299	1.299	0.648
1SPR	1.254	2.252	1.732	0.753	2.035	4.963	2.79	1.32
1Y0M	0.478	1.227	0.67	0.622	0.735	1.898	1.178	0.866
2G6F	0.524	0.916	0.724	0.576	0.9	1.568	1.459	0.686
2DS8	0.979	1.375	1.375	1.191	1.058	1.533	1.533	1.203
1BFE	0.487	0.588	0.588	0.609	0.496	0.621	0.621	0.745
1GFD	1.096	1.398	1.476	1.199	1.37	1.854	1.944	1.249
1EG3	1.343	2.302	1.789	0.656	1.591	2.98	2.219	0.758
1GO5	1.319	2.387	1.109	2.559	1.427	2.531	1.496	2.58
1Z9L	0.701	1.212	1.096	1.278	1.608	2.986	2.735	1.629
2YQL	1.093	1.221	0.914	1.295	1.106	1.283	0.964	1.299
1V49	1.199	1.391	1.247	2.283	1.375	2.324	1.674	2.337
1D1Z	1.548	1.913	1.658	1.146	2.231	3.31	3.18	1.465

† The best complex interface backbone RMSD of the decoys.

§ The best peptide backbone RMSD of the decoys.

‡ The best peptide interface backbone RMSD of the decoys.

¶ The best complex backbone RMSD of the decoys.

**Figure 3:**
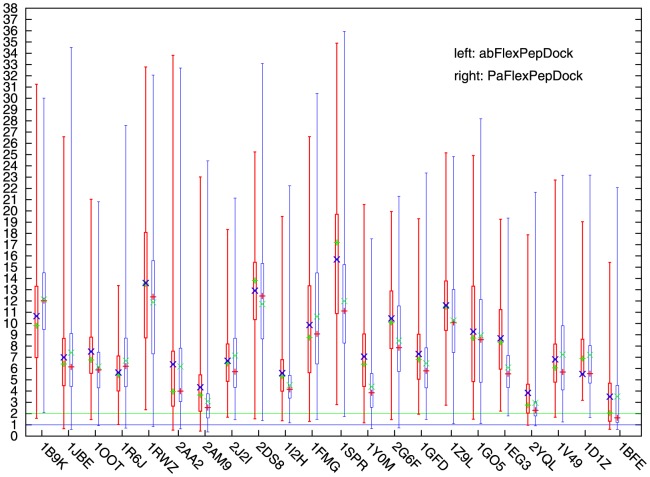
Comparison of PaFlexPepDock and abFlexPepDock on pep_if. Comparison of abFlexPepDock(left) and PaFlexPepDock(right) running with measurement pp_if. ‘+’ stands for mean value, ‘x’ stands for median value. The minimum, lower quartile, upper quartile and maximum are obviously shown without ambiguity. The green horizonal line indicates successful sampling threshold, the blue horizonal line indicates sub-angstrom sampling threshold.

For all of the test cases, PaFlexPepDock achieved successful samplings except for 2AM9, and 11 of the successful samplings had sub-angstrom accuracy. abFlexPepDock failed 4 cases to generate successful samplings, and obtained only 9 sub-angstrom predictions. Thus, PaFlexPepDock performed slightly better than abFlexPepDock when sampling the docking interface. The peptide interface was predicted accurately for 2AM9 (see **Figure 3**) although its com_if was not better. The unsuccessful sampling of com_if was due to the failure of receptor modeling (see the Discussion section for details).

Next, we considered the statistical properties of the decoys obtained by PaFlexPepDock and abFlexPepDock to evaluate predictive accuracy of the peptide interface. **Figure 3** shows the distributions of the decoys on the peptide interface backbone RMSD using abFlexPepDock and PaFlexPepDock by a box-and-whisker plot.

**Figure 4:**
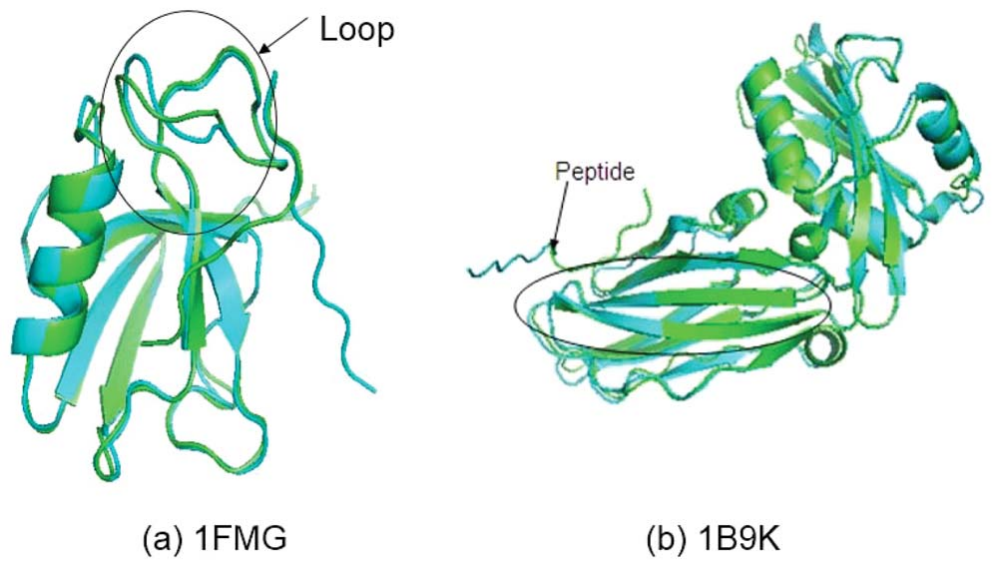
Two failure cases where modeling the flexible regions accurately is hard. For each complex, the start and native pdb were shown in blue and green cartoon,respectively. The decoys generated by PaFlexPepDock protocol were shown in other colors.

The figure shows that the only one case with no successful pep_if sampling was 1B9K. PaFlexPepDock failed to obtain a successful sampling for 1B9K, which was illustrated in **Figure 4** and explained in the Discussion section.

After studying **Figure 3**, we found that for most cases PaFlexPepDock had statistical advantages over abFlexPepDock on mean value,median value,upper quartiles and lower quartiles. The figure illustrated that PaFlexPepDock generally behaved better than abFlexPepDock on pep_if (see also in **Table 2**). Combined analysis of the predictive accuracy for the peptide interface and the complex interface showed that PaFlexPepDock performed better than abFlexPepDock.

Our second part of results is focused on the accuracy of modeling peptide. PaFlexPepDock folds peptides in *ab-initio* manner, so we evaluated how well it worked for free modeling a peptide. Column^¶^ in **Table 3** showed the lowest pp_bb values with PaFlexPepDock and abFlexPepDock (see also in **Table 2**). For 18 out of 22 complexes, PaFlexPepDock produced good models of the peptide (pp_bb less than 2 Å), six of which had a sub-angstrom accuracy. The protocol abFlexPepDock performed worse than PaFlexPepDock. Two particularly successful cases of PaFlexPepDock were 1R6J and 1RWZ, where the peptides contained *β* sheets. The receptor also had a *β* sheet close to the peptide, which formed *β* strands with the peptide. PaFlexDepDock had its lowest pp_bb with 1R6J, which ranged from 1.015 Å (abFlexPepDock) to 0.71 Å (PaFlexPepDock), while for 1RWZ ranged from 2.339 Å (abFlexPepDock) to 0.847 Å (PaFlexPepDock). It is worth mentioning that both cases got sub-angstrom models and the rank of them was relative to the front of the decoys after sorted by energy score. According to the column of difficulty, among the 22 cases only four were classified into easy level, it means that most of the peptides had large difference between its start and native structure.

**Table 3:** The comparison of modeling peptide between PaFlexPepDock and abFlexPepDock.

demo_info		PaFlexPepDock(Å)			abFlexPepDock(Å)		
pdb_id	difficulty†	near_native§	best_location‡	best_pp_bb¶	near_native§	best_rmsd‡	best_pp_bb¶
1B9K	D	none	2%	2.07	14	22%	1.599
1JBE	D	21	26%	0.618	12	5%	0.741
1OOT	D	15	1%	1.737	none	33	2.8
1R6J	M	1	4%	0.71	1	1%	1.015
1RWZ	D	68	16%	0.847	none	74%	2.339
2AA2	D	367	4%	1.088	64	41%	1.644
2AM9	D	2	15%	0.448	2	1385	0.489
2J2I	E	111	50%	1.494	18	18	1.576
1I2H	M	45	22%	1.289	13	7%	1.371
1FMG	D	240	2%	1.495	5	15%	1.299
1SPR	M	none	25%	2.252	none	30%	4.963
1Y0M	M	16	4%	1.227	12	12	1.898
2G6F	M	1	2%	0.916	32	153	1.568
2DS8	E	3	11%	1.375	15	62%	1.533
1BFE	E	1	44%	0.588	1	19%	0.621
1GFD	M	5	1%	1.398	17	12%	1.854
1EG3	M	none	80%	2.302	none	79%	2.98
1GO5	D	none	4%	2.387	none	53%	2.531
1Z9L	E	33	40%	1.212	none	0.4%	2.986
2YQL	M	84	59%	1.221	812	1%	1.283
1V49	M	664	53%	1.391	none	40%	2.324
1D1Z	M	7261	72%	1.913	none	4%	3.31

† Easy/Medium/Difficult.

§ The rank of the first near native peptide sorted by energy value.

‡ The ratio of the rank of the lowest pp_bb over the size of the decoys.

¶ The best pp_bb.

**Figure 5:**
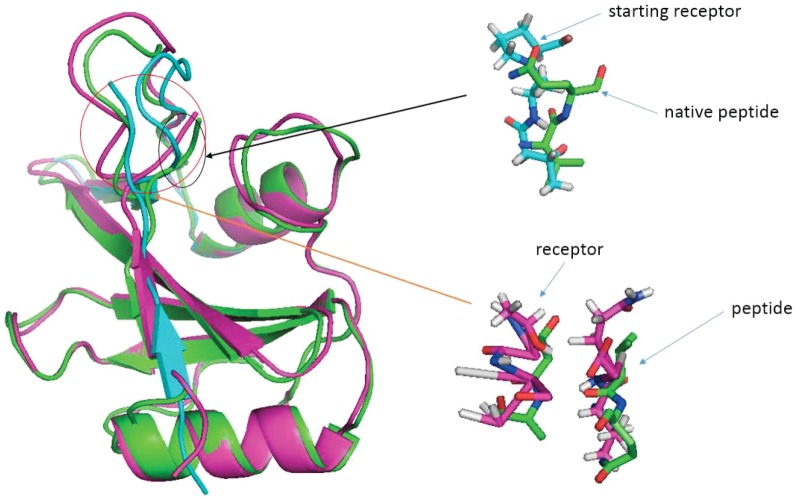
Modeling receptor flexibility contributes to folding peptide. On the left panel,the starting,native and decoy generated by PaFlexPepDock protocol were shown in blue, green and purple cartoon respectively. On the right top panel, the structure conflicts between staring receptor and native peptide were shown in full atom model. The right bottom panel showed the critical contacts between receptor and peptide in full atom model. The green one represents native pose, purple one represents the decoy generated by PaFlexPepDock protocol.

It is not hard to understand that abFlexPepDock was not likely to generate high quality conformation if it treated the receptor as a rigid body. **Figure 5** shows a typical example where the flexible area of receptor is critical to modeling the peptide interface correctly. Thus our protocol with receptor flexibility helps to obtain the accurate peptide and better docking result. After superimposing the starting and native conformation, we can see that the interface of the starting receptor and the peptide is much looser than the native one (carton representation in **Figure 5**). Full investigation showed that the flexible area of the starting receptor collided with the peptide of native (right top in **Figure 5**). That is to say, without backbone movements on receptor, just as what abFlexPepDock did, it is impossible to model correctly the peptide interface. PaFlexDepDock provided a good solution. The modeling peptide and docking peptide to receptor are along with the refining of the receptor flexible areas which enables backbone movements to help peptide folding (left bottom in **Figure 5**). This brought us better chance to obtain near-native conformation.

We also compared PaFlexPepDock with another docking method DynaDock. For these two cases (1BFE and 1GFD), DynaDock obtained best pp_bb values of 1.19 Å and 1.98 Å, respectively, during the first broad sampling stage. The results were improved to 0.66 Å and 1.03 Å after the final refinement stage. PaFlexPepDock produced good result when comparable to those using DynaDock with values 0.588 Å and 1.476 Å, respectively. To obtain insights into the relative success of the sampling and scoring methodologies on peptide backbone RMSD, we used another criteria that was constrained by the best sampled (BS) pose and lowest energy(LE) between PaFlexPepDock and abFlexPepDock.

For those best sampled poses, PaFlexPepDock improved the Ave_rmsd(BS,Å) from 1.447 Å to 1.122 Å and the Ave_rmsd(LE,Å) from 1.929 Å to 1.919 Å(see in **Table 4**). The counts of the BS and LE poses within 2.5 Å pp_bb distance from the bound structure were 22 and five, which were slightly better than abFlexPepDock. A comparison of the results shown in **Table 2** (a summary of results using recently developed protein-peptide docking methods) from ref. [19] showed that PaFlexPepDock was better than some of other docking protocols in terms of the Ave_rmsd. But it was worse than some methods on P(LE), it is our future working to improve it.

**Table 4:** Comparison of the pep_if prediction accuracy constrained by BS and LE.

protocol	P(BS)†	Ave_rmsd(BS)§	P(LE)‡	Ave_rmsd(LE)§
PaFlexPepDock	1	1.122	0.227	1.919
abFlexPepDock	0.727	1.447	0.09	1.929

† P(BS,2.5 Å) the probability of best sampled poses of a docking method being within 2.5 Å pp_bb RMSD of the corresponding native poses.

‡ P(LE,2.5 Å) the probability of lowest energy poses of a docking method being within 2.5 Å pp_bb RMSD of the corresponding native poses.

§ Ave_rmsd(BS,Å) and Ave_rmsd(LE,Å) are the mean value of pp_bb constrained by BS and LE respectively.

**Figure 6:**
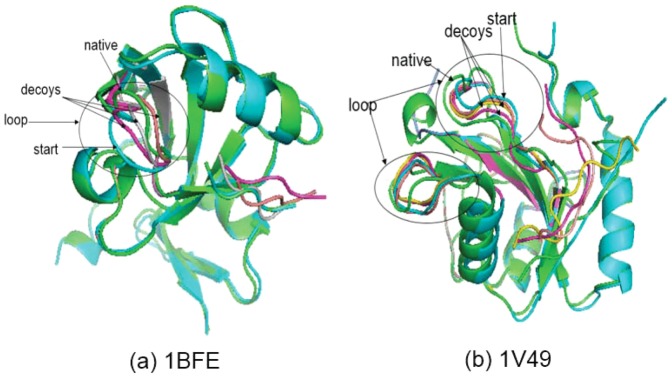
Two successful examples where the receptors had flexible regions. For each complex, the start and native pdb were shown in blue and green cartoon,respectively. The decoys generated by PaFlexPepDock protocol were shown in other colors.

As the third part of our results, we evaluate the performance when modeling the receptor. In most of the examples, the predictive accuracy of the receptor was improved as expected because we explicitly refined the flexible areas of the receptors. **Figure 6** showed two successful examples. The flexible areas are correctly modeled by applying right loop refinement protocol.

**Figure 6** clearly illustrates the flexible regions between the start and the native conformation. The peptides in these two examples were short, so both PaFlexPepDock and abFlexPepDock could fold the peptides to obtain near-native results. However, PaFlexPepDock modeled the receptor more accurately because the appropriate refinement protocol was employed.

Next, we investigated the accuracy of the flexible areas and their relationships to pep_if, which are shown in **Table 5**.

**Table 5:** Comparison of the accuracy of modeling the flexible areas and their relationship to pep_if.

pdb_id	number†	top100#	abFlexPepDock§	PaFlexPepDock§
1B9K	0	4.187	0.80/0.76/0.78	0.38/0.30/0.45
1JBE	4	1.072	0.73	0.57
1OOT	27	0.933	1.12/0.66	0.67/0.5
1R6J	2	1.54	0.80	0.61
1RWZ	28	0.975	0.97	0.97
2AA2	7	0.942	1.86	0.96
2AM9	38	0.766	3.52	2.97
2J2I	0	2.641	1.05	1
1I2H	59	1.307	3.94/1.31	3.58/0.6
1FMG	1	1.495	1.19/0.38	0.52/0.25
1SPR	1	1.732	1.04	0.52
1Y0M	15	0.67	1	0.78
2G6F	1	0.814	0.54/0.80	0.31/0.45
2DS8	2	1.735	0.88	0.69
1BFE	57	0.765	2	1.01
1GFD	48	2.766	0.88	0.69
1EG3	9	1.789	0.17	0.13
1GO5	0	2.531	1.58/2.56	1.38/1.69
1Z9L	2	1.096	3.71	0.47
2YQL	69	0.914	1.48/1.77	0.98/1.15
1V49	1	1.777	2.69/2	1.88/1.33
1D1Z	0	3.74	2.67	0.89

† number: The total number of near-native conformation generated by protocol PaFlexPepDock among top 100 decoys which sorted by flexible regions.

# top100: The lowest value of pep_if among top 100 decoys sorted by flexible regions.

§ The lowest backbone RMSD of flexible regions (each flexible area is evaluated separately).

It was not surprising that for all the 22 examples, PaFlexPepDock predicted the flexible areas more accurately than abFlexPepDock (see column^§^ in **Table 5**). For cases such like 1SPR, and 1D1Z, where there were big difference between starting and native structure on the receptor, PaFlexPepDock reduced much of the backbone RMSD in the flexible areas.

During docking procedure the flexility of receptor connected with peptide interface, so the ability from receptor flexibility to choose peptide interface is very important. In order to find the correlation between these, we gave the lowest value of pep_if among top 100 decoys,after sorting the accuracy of receptor flexible areas in **Table 5**. From **Table 5** we found that there were 18 cases with near-native conformation, even eight get sub-angstrom. For cases like 1I2H and 2YQL, there were even more than half of decoys got near-native peptide interface.

For the last part of our results, we investigated how our sampling policy was related to the energy functions we used. When modeling flexible areas, PaFlexPepDock used the Rosetta full-atom energy function score12 (Table S3 in File S1 shows each energy item) and the coarse grained energy function, which employs a unified spheres side chains model (Rosetta centroid score4) [20]. However, during the post-processing of decoys, abFlexPepDock used the re-weighted energy function include total energy, interface energy, and peptide energy proposed in ref. [7], which showed that 64% of the unbound docking cases in the top-100 models might have a near-native conformation. This was very effective as an energy function for identifying good prediction from the decoys. For PaFlexPepDock, 82% of the top-100 models contained near-native conformations based on this benchmark.

**Figure 7:**
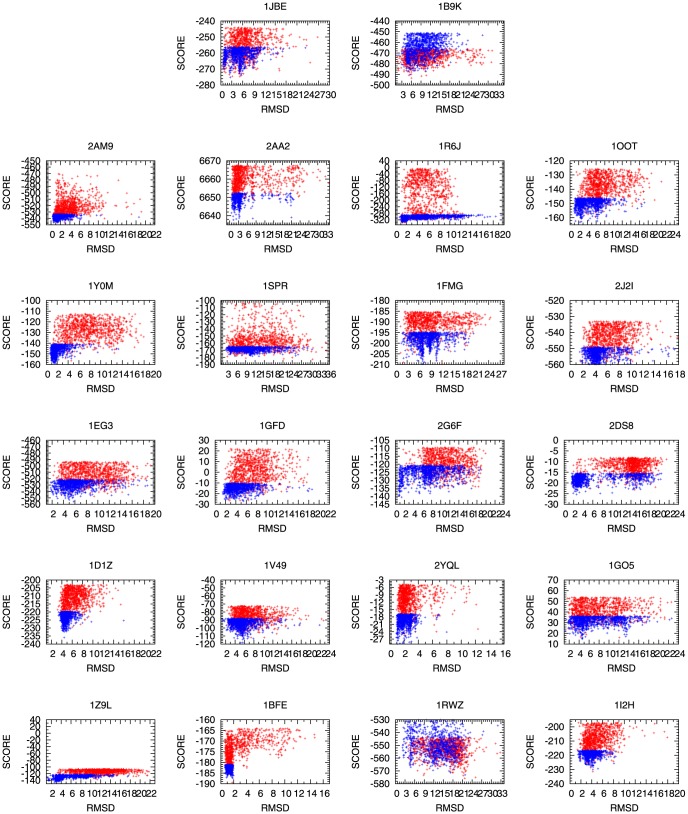
Correlations between the energy value and RMSD. The x-axis and y-axis represent for peptide interface backbone RMSD and re-weight energy score respectively. The points in red color are for abFlexPepDock, the points in blue color are for PaFlexPepDock.

For 19 of 22 cases, PaFlexPepDock produced an excellent energy funnel (e.g.1Y0M,1EG3, and 2YQL). **Figure 7** shows how the peptide interface RMSD was related to the energy function for the test examples. For both of PaFlexPepDock and abFlexPepDock, we chose the models with the lowest 1000 re-weight score values to plot the figure. The blue and red points show the correlation between energy function and peptide interface RMSD created by protocol PaFlexPepDock and abFlexPepDock respectively. For only three cases(1B9K, 1D1Z, and 1FMG), PaFlexPepDock failed to show the energy funnel. We consider that this might have been attributable to the parallel sampling approach that guided energy into the funnels.

### Discussion

PaFlexPepDock combines four samplers in parallel and achieved good performance compared with its predecessor, abFlexPepDock. However, there are two failure cases to be worth discussing here. **Figure 4** shows the two failure cases where PaFlexPepDock had no satisfied performance.

For 1FMG in **Figure 4**, both ends of the flexible regions are connected to *β*-sheets. Both of our loop samplers, backrub and KIC, failed to rotate the unbound flexible segment to the bound position, unlike the successful models shown in **Figure 6**. We think that there might be due to two possible reasons why we could not model these flexible areas accurately. Either the loop sampling was not sufficient or efficient, or the energy function we used rejected good models. Thus, there is a new challenge of modeling flexible loops accurately and efficiently, which will also benefit other modeling applications.

For case 1B9K in **Figure 4**, this is the only one exceptional worse prediction for PaFlexPepDock compared with the target result for abFlexPepDock shown in ref. [7]. In fact, even we rerun abFlexPepDock on that case locally in our computer, we could not obtain the similar results published in ref. [7], while locally redoing of other seven cases would reproduce the similar results in ref. [7]. We believe that was due to using different starting pdb data for this case in this study and in ref. [7]. We tend to think that this only exceptional case did not hurt our conclusion much.

**Figure 8:**
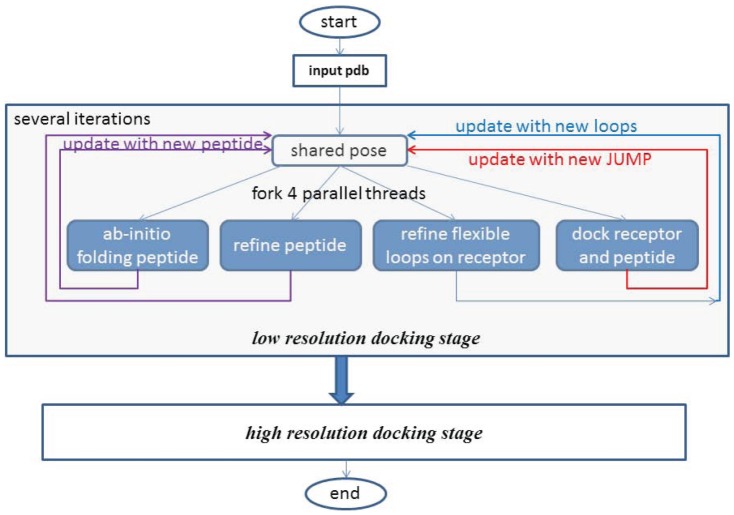
General parallel combination flowchart of PaFlexPepDock.

**Figure 8** shows the ideal parallel design of PaFlexPepDock required to fully share the pose across the four threads. We expect that every single move made by any thread will be sensed by other threads via the shared pose (fine-grained parallelization). Unfortunately, this synchronization will disrupt the consistent data contained in the pose because of the complex design and implementation of Rosetta poses [21]. Thus, we have to make the parallel thread and lock the shared pose while updating occurs. Therefore, from a design level, all four movements of the docking process occur simultaneously, whereas at the implementation level, they occur semi-simultaneously. However, they will behave totally different from that use sequential “simultaneous” movements [7].

PaFlexPepDock assumes that the protein-peptide binding site is known approximately, in the same way as abFlexPepDock. Indeed, binding site prediction can be treated in the same way as other computational problems [22] that involves vast amounts of information from cross-linking experiments, mutational analysis, NMR shifts, or any other experimental evidence [23], [24]. Thus, the identification of flexible areas in this study is also relied on the binding site of the bound structure. Applying an automatic approach [25] alone is not sufficient for locating these flexible areas.

In order to investigate how much contributions each parallel move makes, we conducted a pilot experiment for the prediction procedure of testing case 1D1Z. We collected how frequent each parallel move updates the shared pose (see Table S4 in File S1). The ab-initial peptide folding protocol contributed most to the shared pose 99% out of its moves are updated to the shared pose. Peptide refinement protocol contributed 16% out of its moves to the shared pose. It is not surprising that these protocols made most significant changes on the complex, because the peptide is folded from an extended segment. The modeling flexible protocol offered 13.12% out of its moves to the shared pose, which was much higher than the docking protocol. Notice that all the four protocols are running in parallel. So the updates to the shared pose are broadcast to all of the protocols. So they are helping each other to improve the shared pose towards the direction of lower energy.

## Methods

PaFlexPepDock was constructed from the previous successful docking protocols in an incremental manner. The first building block was Rosetta platform [6], [26], which is a powerful tool for modeling protein structures [27]–[30]. RosettaLigand [31] and RosettaDock [32] were then built on Rosetta to provide docking services for the protein-ligand and protein-protein complexes, respectively. Next, FlexPepDock [5] was developed based on these docking services to facilitate the modeling of protein-peptide interactions with a limited flexibility receptor and peptide. Furthermore, abFlexPepDock [7] was proposed to enhance FlexPepDock by docking with an initial extended peptide. Finally in this study, we extended abFlexPepDock by not only including an extra refinement step for the flexible areas of the receptor, but also parallelizing all the movements during the docking process.

Similar to the way that abFlexPepDock prepares the input data before docking, PaFlexPepDock randomly selected a residue of the peptide as the anchor. PaFlexPepDock also needs to build a fragment library of the peptide, and to determine the binding site manually. As the new enhancement, PaFlexPepDock must address three more issues: 1) identifying the flexible areas of the receptor, 2) applying perturbations to the flexible areas and 3) parallelizing all of the major activities during docking. Next we explain how these three issues are implemented.

The first issue was how to identify the flexible areas of the receptors. There are some computational approaches to identifying the flexible areas for protein-protein docking [25]. But most previous models usually predefine flexible regions by visually comparing the bound and unbound structures. We used the same strategy to identify the possible flexible areas on the receptor for those protein-peptide docking test instances. First, we collected these residues according to a predefined b-factor cutoff value (≥10) in the bound structure. Next, we located the residues around the peptide within a distance of 5 Å. We then obtained the intersection of these two sets of residues, as described in [25]. Finally, for each candidate residue, we calculated the distance between the residue in the bound structure and that in the unbound one. If the distance exceeded a predefined cutoff value (≥6 Å), the residue was judged to belong to a flexible area. In terms of real *ab-initio* protein-peptide docking approaches, the method we proposed here for identifying flexible areas of the receptor is not actually automatic because we need to know the binding site of the bound structure. As discussed earlier, identifying binding site is another challenge that requires more combination of the computational methods and experimental data, which is beyond the focus of this paper.

The second issue of designing PaFlexPepDock was to find a refinement protocol that could be applied to the flexible areas we identified. We used either the Backrub [33] or Kinematic closure (KIC) protocols [34]. Thanks to Rosetta developers [21], these protocols have already been implemented as the backrub mover and the KIC mover within the Rosetta platform. So PaFlexPepDock can apply them easily to the flexible areas. Backrub rotates a backbone segment after adjusting the positions of all the atoms within this segment, thus can provide realistic, small perturbations to rigid backbones. KIC perturbs several degrees of freedom in a backbone segment and tunes the positions of various critical points to make this segment a valid peptide segment. These movers have different performances on different kinds of areas. Basically in this study, we selected the move for each case according to the motion of the flexible area between the starting and native pose. We applied Backrub mover to cases like 1BFE and 1V49 in **Figure 6** where had obvious and regular flexibility between the native and starting conformation. For other cases where tiny movements were identified, we used KIC mover.

The final issue of implementing PaFlexPepDock was the combination of all the movements into a parallel computing framework. The complete PaFlexPepDock pipeline was divided into two stages: low resolution docking and high resolution docking. We think the refinement of the flexible areas of the receptor might cause larger movements of backbone which will consequently affect docking a folding peptide onto the receptor, so we applied the refinement mover in the low resolution docking stage. Three other movers were also employed in this stage: *ab-initio* peptide folding, refinement of the peptide, and the receptor docking with the peptide.

Using OpenMP [35], a parallel computing environment that runs at the thread level, PaFlexPepDock forked four parallel threads with each binding one of the four movers: folding, peptide refinement, docking and refinement of flexible areas of the receptor. The four movers sampled corresponding degrees of freedom on their private working conformations (*pose*s in Rosetta's terminology). The working pose was copied from a shared pose which was updated after an iteration of each mover running within a thread. In this way, the best-so-far predicted pose of each mover was made available to all the movers by the shared pose. The general flowchart of how to implement PaFlexPepDock is shown in **Figure 8**.

The four parallel movers are running asynchronously in **Figure 8**, which means that the update to the shared pose from each iteration will occur at different time. In fact, some movers might require more CPU time for one iteration, while others need less. We would like to point out that the four movers update the shared pose only using their own working results, depicted in update arrows with different colors in **Figure 8**. The refinement of the flexible areas of the receptor updated the shared pose using only the new coordination of the sampled areas. The docking mover also updated the shared pose using only the relative positional coordinates (the JUMP properties in RosettaDock's terminology [21]) of the protein and peptide. The *ab-initio* peptide folding and refinement movers updated the peptide part of the shared pose using the gradually optimized peptide structure. Thus, the optimized results of each individual mover could be sensed by other movers in the next iteration. When the four parallel threads terminate, the shared pose is the final prediction of PaFlexPepDock.

To maintain the consistency of the data in the shared pose, each modification from the parallel thread to the shared pose will be exclusively updated. This was achieved easily by using the lock mechanism provided by OpenMP. To make CPU more efficient, we only allocated two CPU cores for the four parallel threads.

After PaFlexPepDock generated decoys, we use the same clustering approach as abFlexPepDock to find the best predictions from the decoys. How to select the correct model from all the computer generated models is another challenge. We first clustered our computational models using the Rosetta Cluster application, as described in ref. [32], with a cluster radius cutoff of 2 Å. Then we selected a representative model according to the lowest energy score from each cluster. At the same time, the clusters were ranked according to the energy of their representative models.

## Supporting Information

File S1 **Supporting Information.** **Table S1:**The dataset we used in this study includes 22 Unbound protein peptide complex structures. **Table S2:** Statistical comparison to case 1Y0M between 10000 decoys and 50000 decoys on pp_if. **Table S3:** Energy Score. **Table S4:** The update frequency on each thread.
(DOCX)
