# Correction: PaFlexPepDock: Parallel Ab-Initio Docking of Peptides onto Their Receptors with Full Flexibility Based on Rosetta

**DOI:** 10.1371/journal.pone.0105715

**Published:** 2014-08-12

**Authors:** The PLOS ONE Staff

The first 8 values in column 3 of Table 1 are incorrect. The authors have provided a corrected version of Table 1 here.

**Table 1:** The discriminative ability comparison between PaFlexPepDock and abFlexPepDock.

pdb_id	best pep_if †		top-10 pep_if§		First near native cluster¶	
1B9K	2.086	1.581	5.642	1.4	*>* 500	8
1JBE	0.598	0.634	0.96	5	2	29
1OOT	0.933	1.467	1.258	3.2	3	22
1R6J	0.71	1.015	1.9	1.9	1	1
1RWZ	0.847	2.339	4.281	4.3	136	*>* 500
2AA2	0.646	0.532	1.6	1.5	4	10
2AM9	0.362	0.439	0.794	2.6	1	28
2J2I	1.454	1.672	2.759	3.7	4	299
1I2H	1.179	1.363	2.393	1.98	1	1
1FMG	1.495	1.299	5.903	2.025	18	8
1SPR	1.732	2.79	3.887	3.054	205	*>* 500
1Y0M	0.67	1.178	0.869	1.226	1	19
2G6F	0.724	1.459	1.372	3.774	1	25
2DS8	1.375	1.533	1.892	2.471	8	8
1BFE	0.588	0.621	1.314	1.353	1	1
1GFD	1.476	1.944	2.036	2.513	2	150
1EG3	1.789	2.219	3.922	3.603	42	*>* 500
1GO5	1.109	1.496	2.314	3.977	1	28
1Z9L	1.096	2.735	2.984	3.331	12	*>* 500
2YQL	0.914	0.964	1.403	1.46	1	1
1V49	1.247	1.674	3.79	4.917	191	146
1D1Z	1.658	3.18	4.086	4.208	155	*>* 500

Cluster performance of peptide modeling onto unbound protein receptor structures. For each pair, left and right column are generated by PaFlexPepDock and abFlexPepDock respectively.

*^†^*The best pep_if among all sampled decoys.

*^§^*The best pep_if of the representing prediction among top-10 clusters.

¶ The rank of the first cluster with near native structure.
