# Supplementary material for: PaFlexPepDock: Parallel Ab-Initio Docking of Peptides onto Their Receptors with Full Flexibility Based on Rosetta
Source: PLoS One. 2014 May 6;9(5):e94769. doi: 10.1371/journal.pone.0094769 (PMC4011740; doi:10.1371/journal.pone.0094769)
Supplement: File S1 — Supporting Information. Table S1:The dataset we used in this study includes 22 Unbound protein peptide complex structures. Table S2: Statistical comparison to case 1Y0M between 10000 decoys and 50000 decoys on pp_if. Table S3: Energy Score. Table S4: The update frequency on each thread. (DOCX) [file pone.0094769.s001.docx]

## Table S1: The dataset we used in this study includes 22 Unbound protein peptide complex structures

| Unbound/bound*^a^* | Anchor*^b^* | Start_bb/start_ phipsi/start_if*^c^* | Flexible area*^d^* | Loop mover*^e^* | | Length*^f^* | 2^nd^ strc. *^g^* |
| --- | --- | --- | --- | --- | --- | --- | --- |
| 1B9K/2VJ0 | 242 | 17.092/72.173/10.107 | 15-21,  44-51,  69-85 | KIC | 8 | | c |
| 1JBE/2FMF | 129 | 16.123/130.99/11.544 | 86-92 | backrub | 13 | | α |
| 1OOT/1SSH | 67 | 8.356/49.591/4.304 | 10-17,  31-38 | backrub | 11 | | c |
| 1R6J/1W9E | 167 | 4.682/38.905/2.51 | 90-99 | KIC | 5 | | β + c |
| 1RWZ/1RXZ | 247 | 8.861/80.43/5.512 | 115-119,  239-242 | backrub | 11 | | β + c +α |
| 2AA2/2A3I | 260 | 16.382/114.694/10.128 | 21-33 | KIC | 12 | | α+ c |
| 2AM9/1T7R | 254 | 11.52/128.227/5.663 | 220-229 | backrub | 10 | | α |
| 2J2I/2C3I | 270 | 3.231/70.304/1.198 | 209-212 | backrub | 8 | | c |
| 1I2H/1DDV | 107 | 5.172/54.401/3.392 | 10-19,  63-68 | backrub | 6 | | c |
| 1FMG/2H3L | 93 | 12.014/62.367/6.954 | 20-25,  35-39 | backrub | 9 | | c |
| 1SPR/1SPS | 106 | 4.456/45.549/4.443 | 61-67 | backrub | 7 | | c |
| 1Y0M/1YWO | 59 | 4.83/52.961/2.072/ | 29-34 | backrub | 10 | | c |
| 2G6F/2AK5 | 57 | 4.717/47.843/2.708 | 9-13,  25-33 | backrub | 8 | | c |
| 2DS8/2DS7 | 43 | 1.805/59.872/1.2 | 17-25 | backrub | 6 | | c |
| 1BFE/1BE9 | 118 | 0.94/17.245/0.595 | 14-22 | backrub | 5 | | c |
| 1GFD/1IO6 | 64 | 5.643/73.141/3.913 | 30-36,  40-46 | KIC | 10 | | c |
| 1EG3/1EG4 | 267 | 4.819/70.128/2.182 | 76-82 | backrub | 13 | | c |
| 1GO5/1OAI | 63 | 8.782/77.275/4.936 | 29-34,  45-54 | backrub | 9 | | c |
| 1Z9L/1Z9O | 123 | 3.011/55.832/2.087 | 69-83 | backrub | 9 | | c |
| 2YQL/2PUY | 53 | 6.193/26.795/2.331 | 5-12,  27-39 | backrub | 10 | | b+c |
| 1V49/2ZJD | 125 | 4.533/69.152/2.587 | 35-50,  70-79 | backrub | 10 | | c |
| 1D1Z/1D4T | 106 | 4.541/55.022/2.502 | 62-73 | backrub | 10 | | b+c |

*^a^* unbound (start) and bound (native) pdb code respectively.

*^b^* anchor residue of receptor for docking an extended peptide.

*^c^* difference between starting and native structures measured in C α RMSD and pair (ϕ, ψ) deviation respectively.

*^d^* flexible areas on receptor represented with loops segments.

*^e^* refinement protocol used for flexible areas.

*^f^* residue number of peptide to dock.

*^g^* second structure of the peptide.

## Table S2: Statistical comparison to case 1Y0M between 10000 decoys and 50000 decoys on pp_if

| Term | 10000-decoys | 50000-decoys |
| --- | --- | --- |
| lowest-value | 0.67 | 0.599 |
| lower quartiles | 2.561 | 2.561 |
| median value | 3.847 | 3.82 |
| upper quartiles | 5.566 | 5.51 |
| highest-value | 17.515 | 19.622 |
| mean value | 4.3873119 | 4.34315178 |

## Table S3: Energy Score

| Score Term | Weight | Description |
| --- | --- | --- |
| fa_atr | 0.8 | lennard-jones attractive |
| fa_rep | 0.44 | lennard-jones repulsive |
| fa_sol | 0.65 | lazaridis-jarplus solvation energy |
| fa_intra_rep | 0.004 | lennard-jones repulsive between atoms in the same residue |
| fa_pair | 0.49 | pairwise electrostatics term derived from statistics on the pdb database |
| fa_plane | 0 | pi-pi interaction between aromatic groups, by default = 0 |
| fa_dun | 0.56 | internal energy of sidechain rotamers as derived from Dunbrack's statistics |
| ref | 1 | reference energy for each amino acid |
| hbond_lr_bb | 1.17 | long range (beta or loop) backbone-backbone hydrogen bonds |
| hbond_sr\bb | 0.585 | short range (helix) backbone-backbone hbonds |
| hbond_bb_sc | 1.17 | sidechain-backbone hydrogen bond energy |
| hbond_sc | 1.1 | sidechain-sidechain hydrogen bond energy |
| p_aa_pp | 0.32 | Probability of amino acid at phipsi |
| dslf_ss_dst | 0.5 | distance score in current disulfide |
| dslf_cs_ang | 2 | csangles score in current disulfide |
| dslf_ss_dih | 5 | dihedral score in current disulfide |
| dslf_ca_dih | 5 | ca dihedral score in current disulfide |
| pro_close | 1 | proline ring closure energy |
| rama | 0.2 | ramachandran preferences |
| omega | 0.5 | omega dihedral in the backbone |

## Table S4: The update frequency on each thread

| Item | Receptor refine | Peptide abinitio | Peptide refine | docking |
| --- | --- | --- | --- | --- |
| accept | 328 | 2492 | 417 | 145 |
| reject | 2182 | 18 | 2093 | 2365 |
| share rate | 13.12% | 99.68% | 16.68% | 5.8% |
